# Intraindividual comparison of [^177^Lu]Lu-DOTA-EB-TATE and [^177^Lu]Lu-DOTA-TOC

**DOI:** 10.1007/s00259-020-05177-z

**Published:** 2021-01-15

**Authors:** Heribert Hänscheid, Philipp E. Hartrampf, Andreas Schirbel, Andreas K. Buck, Constantin Lapa

**Affiliations:** 1 Department of Nuclear Medicine, University Hospital Würzburg, Oberdürrbacher Str. 6, 97080 Würzburg, Germany; 2 Department of Nuclear Medicine, University Hospital Augsburg, Augsburg, Germany

**Keywords:** DOTA-EB-TATE, Somatostatin receptor, Evans blue, Biokinetics, Intraindividual comparison

## Abstract

**Purpose:**

The radiolabelled somatostatin analogue [^177^Lu]Lu-DOTA-EB-TATE binds to albumin via Evans blue, thereby increasing the residence time in the blood and potentially allowing more therapeutic agent to be absorbed into the target tissue during peptide receptor radionuclide therapy. It was tested in selected patients whether the substance is superior to [^177^Lu]Lu-DOTA-TOC.

**Methods:**

Activity kinetics in organs and tumours after [^177^Lu]Lu-DOTA-EB-TATE and [^177^Lu]Lu-DOTA-TOC were compared intraindividually in five patients with progressive somatostatin receptor-positive disease scheduled for radionuclide therapy.

**Results:**

In comparison to [^177^Lu]Lu-DOTA-TOC, tumour doses per administered activity were higher for [^177^Lu]Lu-DOTA-EB-TATE in 4 of 5 patients (median ratio: 1.7; range: 0.9 to 3.9), kidney doses (median ratio: 3.2; range: 1.6 to 9.8) as well as spleen doses (median ratio: 4.7; range 1.2 to 6.2) in all patients, and liver doses in 3 of 4 evaluable patients (median ratio: 4.0; range: 0.7 to 4.9). The tumour to critical organs absorbed dose ratios were higher after [^177^Lu]Lu-DOTA-TOC in 4 of 5 patients.

**Conclusions:**

Prior to a treatment with [^177^Lu]Lu-DOTA-EB-TATE, it should be assessed individually whether the compound is superior to established substances.

## Introduction

High expression of somatostatin receptors allows for imaging and peptide receptor radionuclide therapy (PRRT) of neoplasms using radiolabelled somatostatin analogues like ^90^Y- and ^177^Lu-labelled [DOTA^0^,Tyr^3^]-octreotate (DOTA-TATE) or -octreotide (DOTA-TOC) [1–4]. Recently, the randomised, controlled NETTER-1 trial demonstrated the efficacy and safety of [^177^Lu]Lu-DOTA-TATE PRRT in patients with well-differentiated, metastatic midgut neuroendocrine tumours (NET) that had progressed under first-line therapy with unlabelled somatostatin analogues [5].

A major problem regarding the tumour absorbed dose in PRRT with DOTA-TATE or DOTA-TOC, however, is the rapid elimination of the active compound from the blood stream [6, 7], eventually leading to insufficient tumour uptake and thus poorer clinical outcome. Assuming first-order kinetics, the uptake into the tumour tissue is expected from pharmacokinetics to be proportional to the time integral of the active compound concentration in the blood. With rapid elimination, only a small fraction of the administered medication flows through the target tissue and can be retained; most of the active compound disappears without reaching the tumour tissue. As a further optimization of PRRT, octreotate bound to Evans blue (DOTA-EB-TATE) has recently been introduced [8]. Evans blue reversibly binds to endogenous albumin, retains octreotate in the blood, and thus extends the effective plasma half-life of the radiolabelled vector considerably. Since somatostatin receptors are still able to bind octreotate held by Evans blue, achievable tumour uptake and absorbed dose might be significantly increased and result in outcomes that are even more favourable. In pilot studies [9, 10] and dose escalation studies [11, 12] in patients with advanced metastatic NET, [^177^Lu]Lu-DOTA-EB-TATE exhibited higher tumour retention and was more effective than [^177^Lu]Lu-DOTA-TATE. Although significantly increased tracer accumulation in the kidneys and red marrow was observed [9], repeated treatment with up to 3.97 GBq/cycle was well tolerated [12].

To offer selected patients the opportunity to benefit from this innovation, we invited them to perform PRRT with the dosimetrically superior compound after an intraindividual comparison of the kinetics of [^177^Lu]Lu-DOTA-EB-TATE and [^177^Lu]Lu-DOTA-TOC. Since no direct intraindividual comparison of the novel compound with established somatostatin analogues has been published so far, we communicate our experience with five patients scheduled for PRRT.

## Material and methods

This report presents the results of intraindividual comparisons of activity kinetics in organs and tumours after intravenous administration of both [^177^Lu]Lu-DOTA-EB-TATE and [^177^Lu]Lu-DOTA-TOC in patients with progressive somatostatin receptor positive malignancies. Patients planned for PRRT, whose risk was considered high prior to treatment, were offered pre-therapeutic kinetics studies to identify the therapeutic agent with the superior tumour to kidney dose ratio. In order to avoid potential side effects due to repeated administration of amino acids, the dosimetric assessments were performed without kidney protection medication.

### Patients

Two female and three male patients (1 malignant pheochromocytoma, 4 neuroendocrine tumours) decided to participate in the measurements. Information on patient characteristics is provided in Table 1. While 3 individuals had no prior PRRT, 2 were scheduled for an additional treatment cycle after 4 preceeding cycles with about 7 GBq [^177^Lu]Lu-DOTA-TOC each. Receptor expression was confirmed in all patients by somatostatin receptor directed positron emission tomography performed no more than 2 months prior to the kinetics study. Peak standardised uptake values in the 1 mL spherical volumes with the highest activity uptakes in positron emission tomography ranged from 17 to 54.

**Table 1:** Patient characteristics

	Patient 1	Patient 2	Patient 3	Patient 4	Patient 5
Sex	M	M	F	M	F
Age	76 y	53 y	51y	55 y	57 y
Weight	76 kg	74 kg	62 kg	90 kg	57 kg
Disease	Ileum NET	Pheochromocytoma	Pancreas NET	Rectum NET	Ileum NET
SUV_peak_	17	29	54	54	22
[^177^Lu]Lu-DOTA-EB-TATE	185 MBq	160 MBq	191 MBq	206 MBq	189 MBq
[^177^Lu]Lu-DOTA-TOC	200 MBq	201 MBq	202 MBq	207 MBq	206 MBq
Previous PRRT cycles	4	0	4	0	0
Previous [^177^Lu]Lu-DOTA-TOC	27 GBq	0	28 GBq	0	0
GFR in ml/min/1.73qm	51	44	103	98	85

*SUV*_*peak*_ standardised uptake value in the 1 mL spherical volume with the highest activity uptake in somatostatin receptor directed positron emission tomography; *GFR* glomerular filtration rate

The additional diagnostic procedures were performed in accordance with §13,2b German Drug Act. All patients signed written informed consent to the activity administrations and the diagnostic procedures as well as to the recording and analysis of their data and the anonymised publication of the results. The local ethics committee expressed no objections to the retrospective evaluation and publication of the data in accordance with data protection regulations (reference number 20200803 01).

### Radiopharmaceuticals

The radiosynthesis of [^177^Lu]Lu-DOTA-EB-TATE was carried out in our GMP laboratory with the same operating procedure that is commonly used for dosimetric activities of [^177^Lu]Lu-DOTA-TOC, starting with a solution of 75 μg DOTA-EB-TATE and 7 mg gentisic acid in 525 μl of a 0.4 M sodium acetate buffer solution (pH 5.2). After adding 300 MBq no-carrier-added [^177^Lu]LuCl_3_ (ITG, Garching, Germany; isomeric purity: < 10^−177m^Lu) in 200 μl 0.04 M, the solution was stored in a heating block for 35 min at 100 °C, cooled, transferred into a sterile bench, diluted with 8 ml saline, and passed through a sterile filter (0.22 mm) into a sterile vial. Every single batch of [^177^Lu]Lu-DOTA-EB-TATE, before release, was tested for radiochemical purity by gradient high-performance liquid chromatography and thin layer chromatography. Since the radiochemical yield was > 97% in all cases, further purification was not necessary. Additionally, the product was tested for pH and a bubble point test was performed. Except for a slightly lower initial peptide amount of 50 μg, the labeling and release procedure was identical for [^177^Lu]Lu-DOTA-TOC.

### Measurements and data evaluation

Activity kinetics were analysed in whole body, the kidneys, livers, spleens, and tumourous lesions from whole body scans performed 5 min, 4 h, 1 day, 2 days, and 4 days after the activity administrations. For [^177^Lu]Lu-DOTA-EB-TATE, an additional scan after 9 days, which turned out to be about a physical half-life later than the typically observed retention maximum, was added to account for the retarded kinetics. As it was not necessary to assume effective half-lives longer than the physical half-life to fit time-activity functions after [^177^Lu]Lu-DOTA-EB-TATE, approximately comparable accuracies of the time integrated activities were obtained for both compounds. All camera measurements were performed with the same dual head gamma camera (Siemens Symbia E, Siemens Healthineers, Erlangen, Germany; equipped with medium energy collimators), identical camera settings (matrix: 1024 × 256; 20% window at 208 keV; identical measuring distance), and scan speed (10 cm/min).

**Fig. 1:**
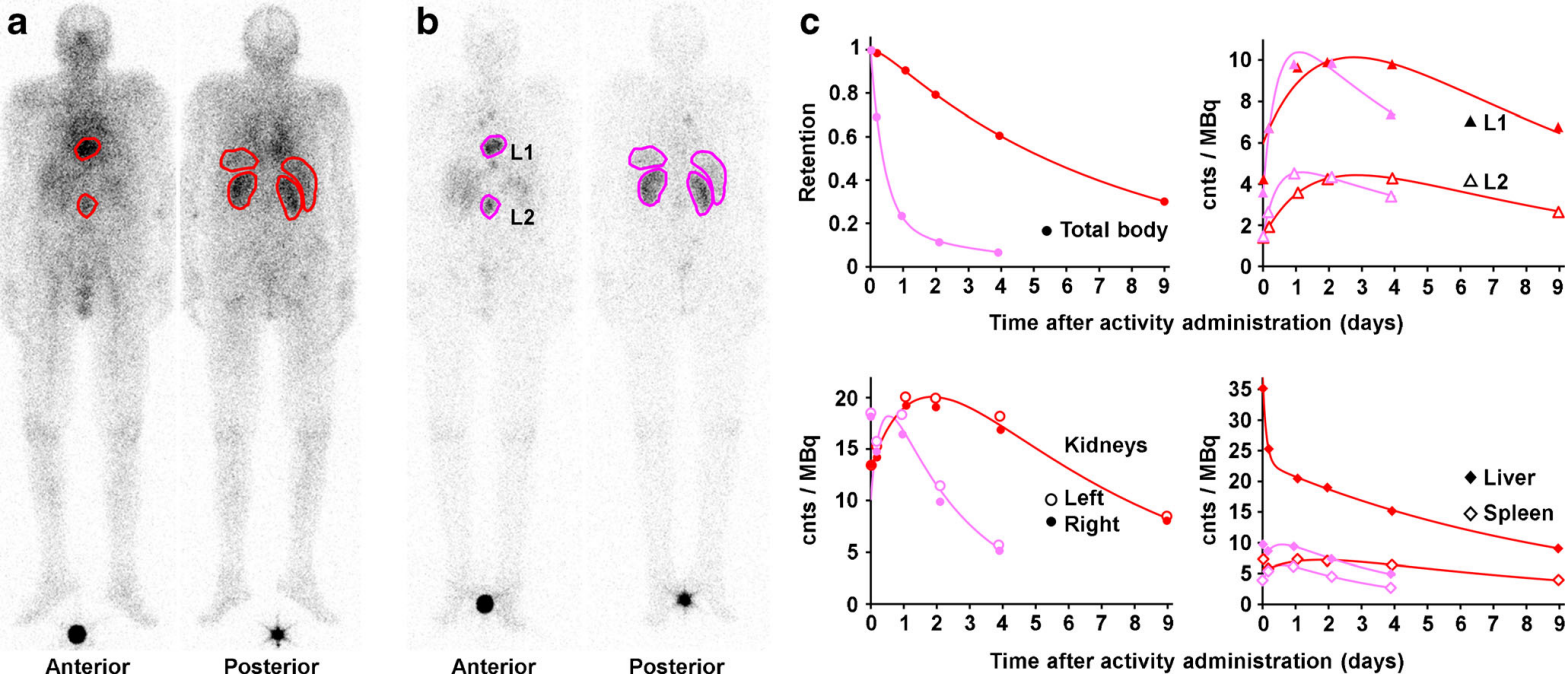
Camera images (with regions of interest; background regions not shown) of Patient 1 taken 1 day after administration of **a** 185 MBq [^**177**^Lu]Lu-DOTA-EB-TATE (red) and **b** 200 MBq [^**177**^Lu]Lu-DOTA-TOC (purple). **c** Measured total body retention and net counts per administered activity (cnts/MBq) in the regions of interest with bi-exponential fit functions. L1 and L2 denote tumour lesions

Regions of interest were drawn including the organ or lesion, or part of the tissue in case of overlapping accumulating tissues, under consideration and over an area with representative background. Identical regions were copied to each scan in both patient series and background-corrected counts were extracted (Fig. 1). No further corrections, e.g., for attenuation or scatter, were applied as these are expected to cancel out in the intraindividual comparison.

The resulting net counts were normalised to the actually administered activity. A bi-exponential decay function was fitted by ordinary least squares regression to the kinetics of net counts per activity and integrated over time (from zero to infinity) to be used as a proxy for the number of decays and thus the absorbed dose in each measured tissue.

Estimates of the absolute absorbed doses per unit administered activity were deduced by normalising the kinetics to activity concentrations measured 2 days after the administration by SPECT/CT (Siemens Intevo Bold, Siemens Healthineers, Erlangen, Germany; equipped with medium energy collimators; reconstructed with xSPECT Quant).

**Fig. 2:**
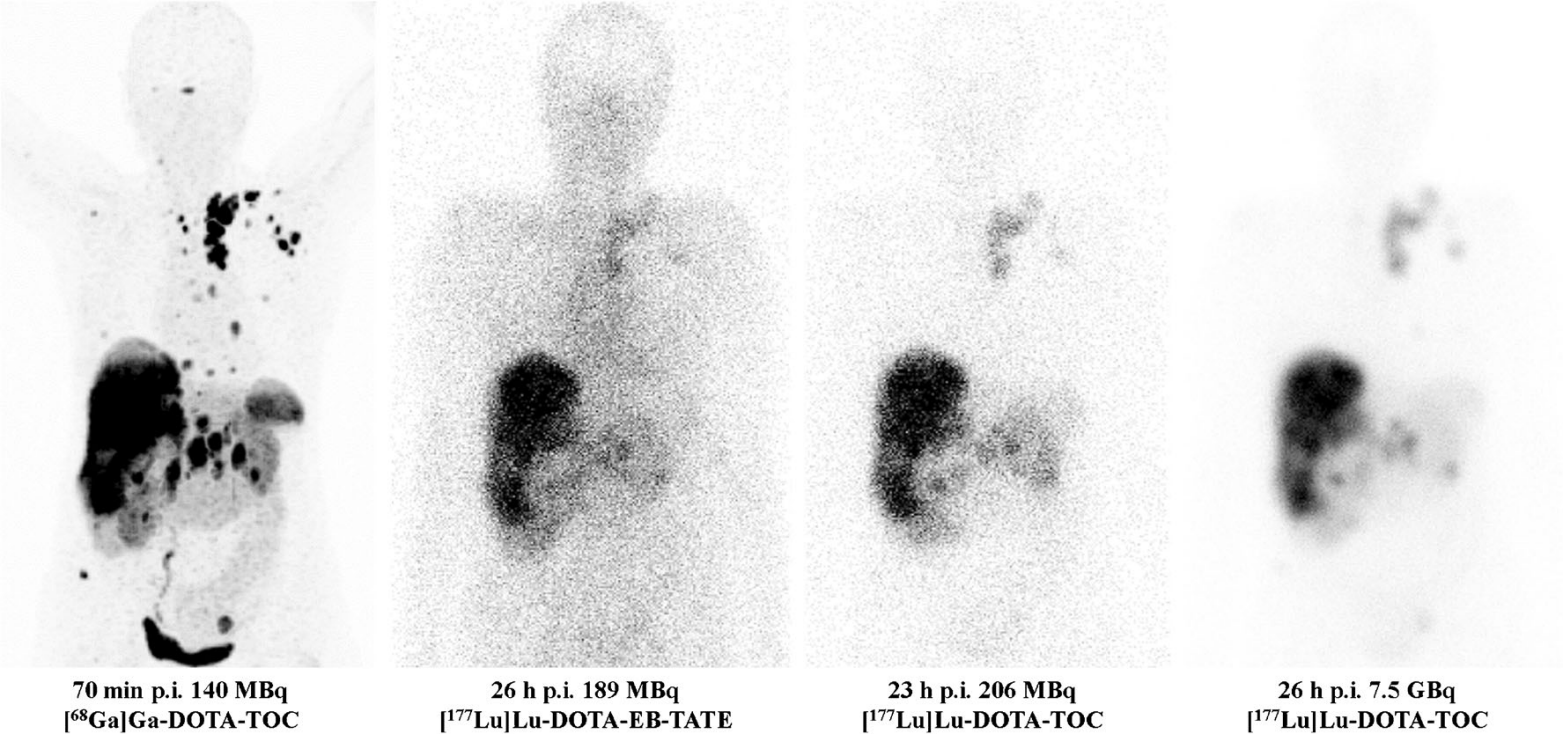
Anterior images of Patient 5 after diagnostics with [^**68**^Ga]Ga-DOTA-TOC (left), [^**177**^Lu]Lu-DOTA-EB-TATE and [^**177**^Lu]Lu-DOTA-TOC (center), and therapy with [^**177**^Lu]Lu-DOTA-TOC (right). The colour scales of the ^**177**^Lu scans are normalised to the activity administered. Tumour to kidneys uptake ratio was identical in both diagnostic assessments with ^**177**^Lu but improved by a factor of 2 with arginine/lysine medication in therapy

Patients 1, 2, and 3 received the second assessment with [^177^Lu]Lu-DOTA-EB-TATE 21, 21, and 15 days after [^177^Lu]Lu-DOTA-TOC, patients 4 and 5 received [^177^Lu]Lu-DOTA-TOC 27 and 21 days after [^177^Lu]Lu-DOTA-EB-TATE, respectively. The counts of the second diagnostic study were corrected for residual counts from the preceding assessment, resulting in less than 5% correction for the kidneys and tumours, with the exception of the liver metastasis in patient 5 (Fig. 2), which prevented an evaluation of the healthy liver tissue in this individual.

In patients 3, 4, and 5, additional blood samples were collected after [^177^Lu]Lu-DOTA-EB-TATE at the time of the scans to determine the absorbed doses to the blood per administered activity from the activity concentrations in whole blood. Blood dose was determined from the time integrated activity coefficients calculated as time integral of a bi-exponential fit function to the blood measurements with the S-value for self-irradiation of blood of 1.72·10^−11^ Gy·ml/(Bq·s) [13].

### Statistical analysis

Deviations of [^177^Lu]Lu-DOTA-EB-TATE to [^177^Lu]Lu-DOTA-TOC dose ratios in Tables 2 and 3 from unity were tested with the Student two-sided one-sample *T* test using JASP [14]. A *p* value <0.05 was considered statistically significant.

**Table 2:** [^**177**^Lu]Lu-DOTA-EB-TATE to [^**177**^Lu]Lu-DOTA-TOC absorbed dose ratios

	Patient 1	Patient 2	Patient 3	Patient 4	Patient 5	Mean	Median	p
Whole body	7.2	4.9	14.8	8.1	3.0	7.6	7.2	0.03
Kidneys	3.2	2.7	9.8	5.1	1.6	4.5	3.2	0.07
Tumours	1.7	3.9	1.7	0.9	1.6	2.0	1.7	0.13
Spleen	4.7	1.2	5.2	6.2	2.8	4.0	4.7	0.03
Liver	3.3	0.7	4.7	4.9		3.4	4.0	0.09

The liver of Patient 5 was not evaluable due to hepatic metastasis

**Table 3:** [^**177**^Lu]Lu-DOTA-EB-TATE to [^**177**^Lu]Lu-DOTA-TOC absorbed dose ratios normalised to the correspondent tumour dose ratios. Values ≥ 1 indicate higher increase in the healthy tissue than in the tumour

	Patient 1	Patient 2	Patient 3	Patient 4	Patient 5	Mean	Median	p
Whole body	4.2	1.3	8.8	9.1	1.9	5.1	4.2	0.07
Kidneys	1.9	0.7	5.9	5.7	1.0	3.0	1.9	0.15
Spleen	2.8	0.3	3.1	7.0	1.7	3.0	2.8	0.15
Liver	1.9	0.2	2.8	5.5		2.6	2.4	0.24

The liver of Patient 5 was not evaluable due to hepatic metastasis

## Results

[^177^Lu]Lu-DOTA-EB-TATE showed high and prolonged retention in blood and an extended accumulation phase with maximum uptake after 2–3 days in most tissues (Fig. 1). The [^177^Lu]Lu-DOTA-EB-TATE to [^177^Lu]Lu-DOTA-TOC absorbed dose ratios, calculated from the ratios of the corresponding time integrals of the bi-exponential decay functions, are shown in Table 2. Tumour doses were higher after [^177^Lu]Lu-DOTA-EB-TATE in 4 of 5 patients (median ratio: 1.7; range: 0.9 to 3.9; p: 0.13), kidney doses in all patients (median ratio: 3.2; range: 1.6 to 9.8; p: 0.07). Ratios were higher in all spleens (median ratio: 4.7; range: 1.2 to 6.2; p: 0.03) and in 3 of 4 evaluable livers (median ratio: 4.0; range: 0.7 to 4.9; p: 0.09).

Normalised to the dose ratios in tumours, the [^177^Lu]Lu-DOTA-EB-TATE to [^177^Lu]Lu-DOTA-TOC absorbed dose ratios (Table 3) were ≥ 1 in whole bodies and organs except for kidneys, spleen, and liver in patient 2, indicating higher increase of doses in healthy tissues than in tumours and thus higher tumour to critical organs absorbed dose ratios for [^177^Lu]Lu-DOTA-TOC. The increase in normalised ratios did not reach significance in any of the tissues.

Time integrated activity coefficients in patients 3, 4, and 5 were 8.0, 6.2, and 6.0 h per litre of whole blood, absorbed doses to blood 0.5, 0.4, and 0.4 Gy per GBq ^177^Lu, respectively.

## Discussion

Evans blue in [^177^Lu]Lu-DOTA-EB-TATE binds the somatostatin analogue DOTA-TATE to albumin and thus protects it from excretion. In fact, the blood residence time was more than one order of magnitude higher than usual for [^177^Lu]Lu-DOTA-TATE [6] in the three patients with blood activity measurements. Further accumulation of activity in tissues such as tumours and kidneys was observed in the 5 patients examined until about 3 days after administration. Potentially, this leads to a higher uptake into the target tissue and an improved therapeutic efficacy [9, 10].

Except in one patient, the number of radioactive decays in tumours per administered activity was indeed increased compared to [^177^Lu]Lu-DOTA-TOC, on average by a factor of 2 (range: 0.9 to 3.9). Unfortunately, the numbers were also increased in the other tissues evaluated, namely the kidneys, spleen, and liver, as well as in the whole body, with numbers almost always higher than in the tumours. Table 4 shows how the increased cumulative activities translate to doses per unit of activity administered. The kidneys are usually the dose-limiting organ in PRRT and only in patient 2 the increase was lower in the kidneys than in the tumours. In contrast to our findings, a 7.9-fold higher tumour dose after [^177^Lu]Lu-DOTA-EB-TATE as compared to [^177^Lu]Lu-DOTA-TATE has recently been reported [9]. However, this has not been deduced intraindividually but from the comparison of observations in distinct patient groups with 5 individuals receiving [^177^Lu]Lu-DOTA-EB-TATE and only 3 controls receiving [^177^Lu]Lu-DOTA-TATE. The present comparison has the advantage that each individual is his or her own control and, due to the identically performed measurements and evaluations, the relative uncertainty of the dose ratios is reduced.

**Table 4:** Absorbed doses per unit administered activity. Due to the uncertainties of quantification in SPECT/CT at low activity levels, the ratios differ from those shown in Table 2

	Patient 1	Patient 2	Patient 3	Patient 4	Patient 5
Gy per GBq [^177^Lu]Lu-DOTA-TOC					
Kidneys	0.75	1.58	0.42	0.67	1.70
Tumours	2.14	0.46	0.64	5.65	4.02
Spleen	0.20	3.06	0.27	0.24	0.39
Liver	0.17	0.49	0.11	0.06	
Gy per GBq [^177^Lu]Lu-DOTA-EB-TATE					
Kidneys	2.63	4.11	3.79	3.73	2.45
Tumours	4.21	1.45	1.17	4.35	6.64
Spleen	1.04	3.85	1.66	1.25	1.10
Liver	0.60	0.39	0.49	0.28	

The liver of Patient 5 was not evaluable due to hepatic metastasis

It should be noted that all measurements were taken without medication for kidney protection. In PRRT with DOTA-TATE or DOTA-TOC, activity is regularly administered to our patients together with an infusion of 25 g of lysine and 25 g of arginine diluted in 2 l of normal saline over 4 h, starting 1 h before PRRT, which significantly reduces renal retention [15]. A direct comparison of the activity kinetics after therapy with the kinetics measured pretherapeutically was not possible, since a complete series of measurements is not scheduled in our operational procedures for a standard therapy with [^177^Lu]Lu-DOTA-TOC. However, an evaluation of the count rates in whole body scans performed 1 day after the activity administration using the same regions of interest as in the dosimetric studies showed that the renal uptake per administered activity was reduced in our patients to an average of 54% (range: 34 to 62%) compared to the corresponding diagnostic assessment. With the accumulation phase extended to 2–3 days, it is not certain that the same treatment will be as effective for DOTA-EB-TATE, or whether the protective medication would need to be extended as well. However, infusion of sufficient amounts of amino acids over a much longer period of time is not indicated due to potentially severe side effects such as persistent nausea and pronounced hyperkalemia.

The second organ that can receive critical doses from PRRT in individual patients is the red bone marrow, which cannot be explained solely by irradiation through blood activity. In our patients, a meaningful measurement of the activity in the bone marrow was not possible due to the low diagnostic activity and the generally high tissue background after [^177^Lu]Lu-DOTA-EB-TATE. Given the high residence time of the activity in the blood, a considerably higher dose to the bone marrow must be expected after [^177^Lu]Lu-DOTA-EB-TATE than the values of 0.03–0.07 Gy/GBq typically calculated for [^177^Lu]Lu-DOTA-TATE and [^177^Lu]Lu-DOTA-TOC [16]. Assuming complete binding of the activity to large plasma proteins and a red marrow-to-blood activity concentration ratio of 0.36 [17] and taking the photon contribution from the remainder of the body into account, OLINDA/EXM [18] indicated absorbed doses to the red marrow of 0.13 to 0.16 Gy/GBq [^177^Lu]Lu-DOTA-EB-TATE for the 3 patients with blood measurements. A considerable release of DOTA-TATE from Evans Blue into the extracellular fluid space would increase the red marrow-to-blood activity concentration ratio and correspondingly the calculated bone marrow absorbed dose. Even higher values might be possible if an existing specific uptake is further enhanced. Although a recent dose escalation study reported no threatening toxicity after 3.7 GBq [^177^Lu]Lu-DOTA-EB-TATE in 14 patients [11], administering activities that would have promised higher doses than those induced by [^177^Lu]Lu-DOTA-TOC would not have been acceptable in our patients.

A major drawback of the present study is the small number of patients. Although the data in Tables 2 and 3 indicate a trend, a conclusive evaluation of the new therapeutic agent is not possible with only 5 individuals included. The increase of the residence time of DOTA-TATE in blood by coupling it to Evans blue is a very interesting approach that should definitely be investigated further, even though our measurements show that certainly not all patients will benefit. None of our patients showed clearly superior kinetics with [^177^Lu]Lu-DOTA-EB-TATE, all 5 individuals included in the present report were treated with the established compound [^177^Lu]Lu-DOTA-TOC. Subsequently, we discontinued the comparative measurements until some open questions are clarified that need to be addressed in prospective trials:

- [^177^Lu]Lu-DOTA-EB-TATE was more effective than [^177^Lu]Lu-DOTA-TATE in the dose escalation study [11], how does it compare at matching doses in kidneys and bone marrow.
- The influence of the Evans blue on the kinetics was quite heterogeneous in our patients, which parameters determine the activity uptake in tumours and organs?
- Which renal protection medication protocol should be used; how effective will it be?
- PRRT is usually applied in several treatment cycles; what are the long-term effects of repeatedly administered high activities of [^177^Lu]Lu-DOTA-EB-TATE?
- How can patients be identified who benefit more from [^177^Lu]Lu-DOTA-EB-TATE as compared to established substances?

Until these questions are answered, we recommend conducting comparative studies of activity kinetics in patients who are being considered for treatment with [^177^Lu]Lu-DOTA-EB-TATE.

## Conclusions

Comparative measurements of activity kinetics in 5 patients showed that the therapeutic index, determined by the ratio of absorbed doses in tumours and critical organs, was not superior for [^177^Lu]Lu-DOTA-EB-TATE compared to [^177^Lu]Lu-DOTA-TOC. Before using [^177^Lu]Lu-DOTA-EB-TATE for PRRT, patient safety and superiority over established substances should be assessed individually.
